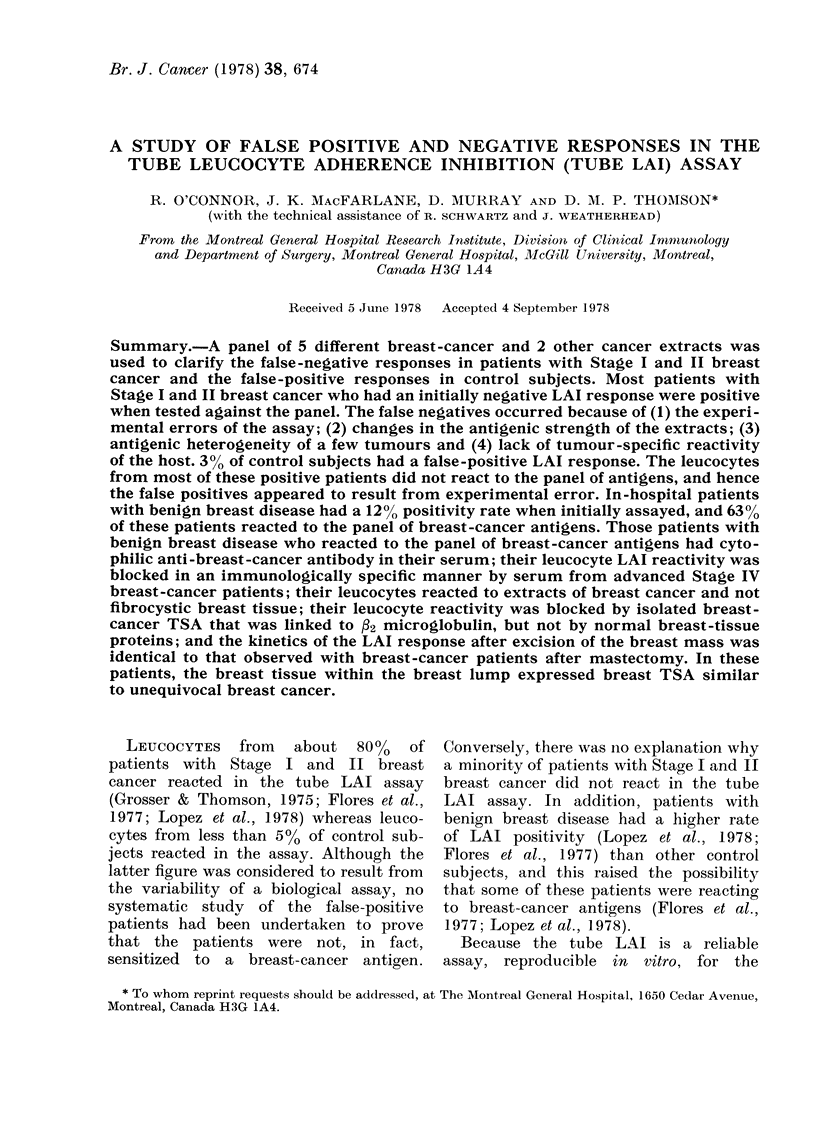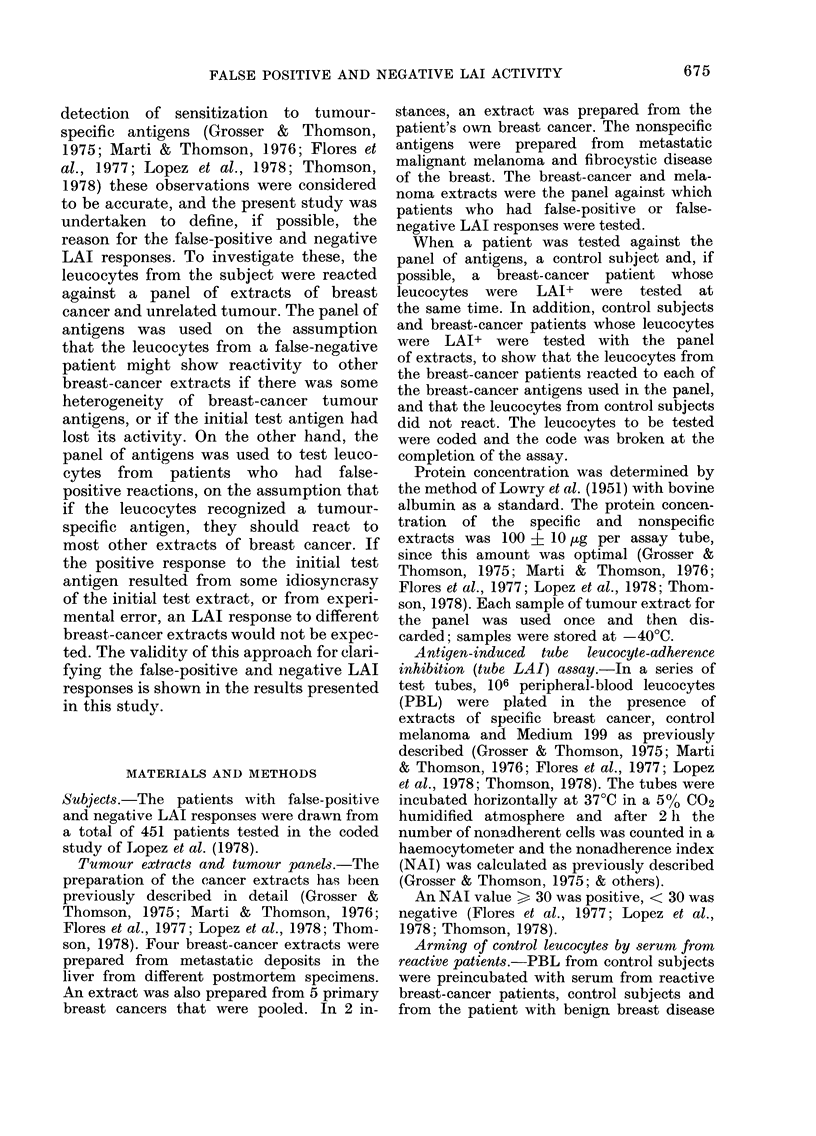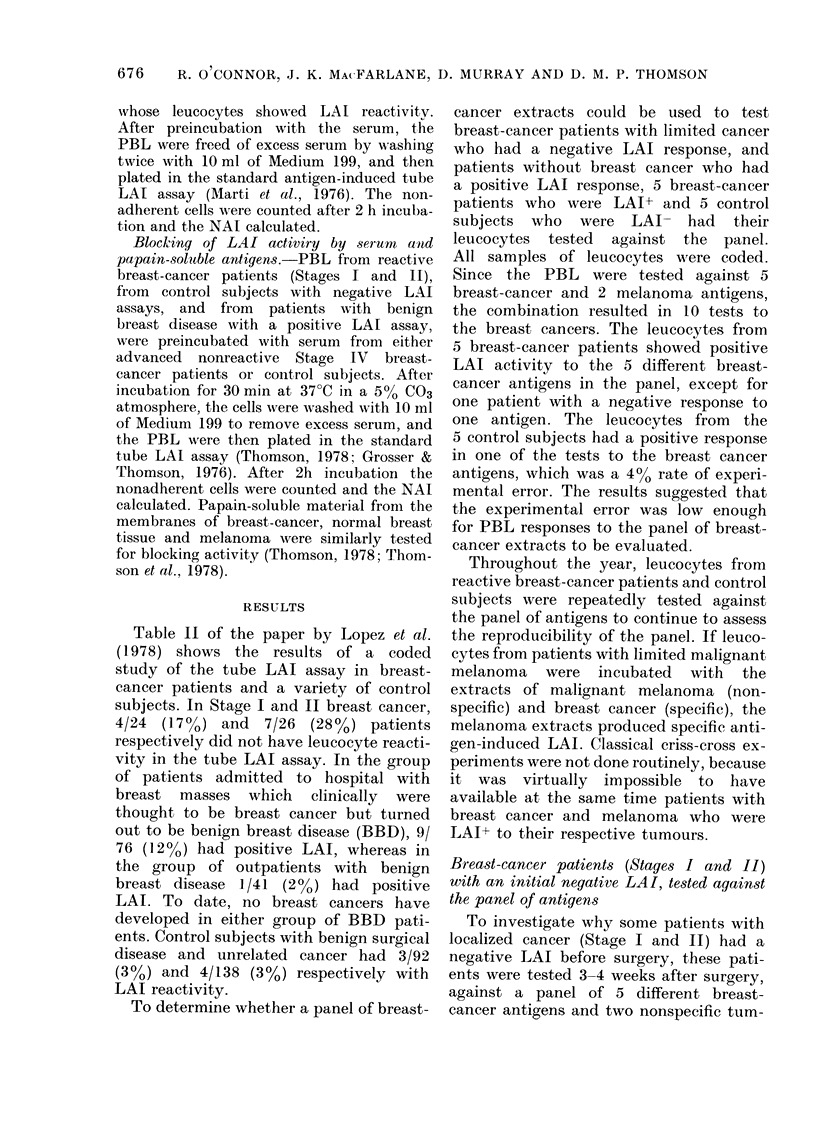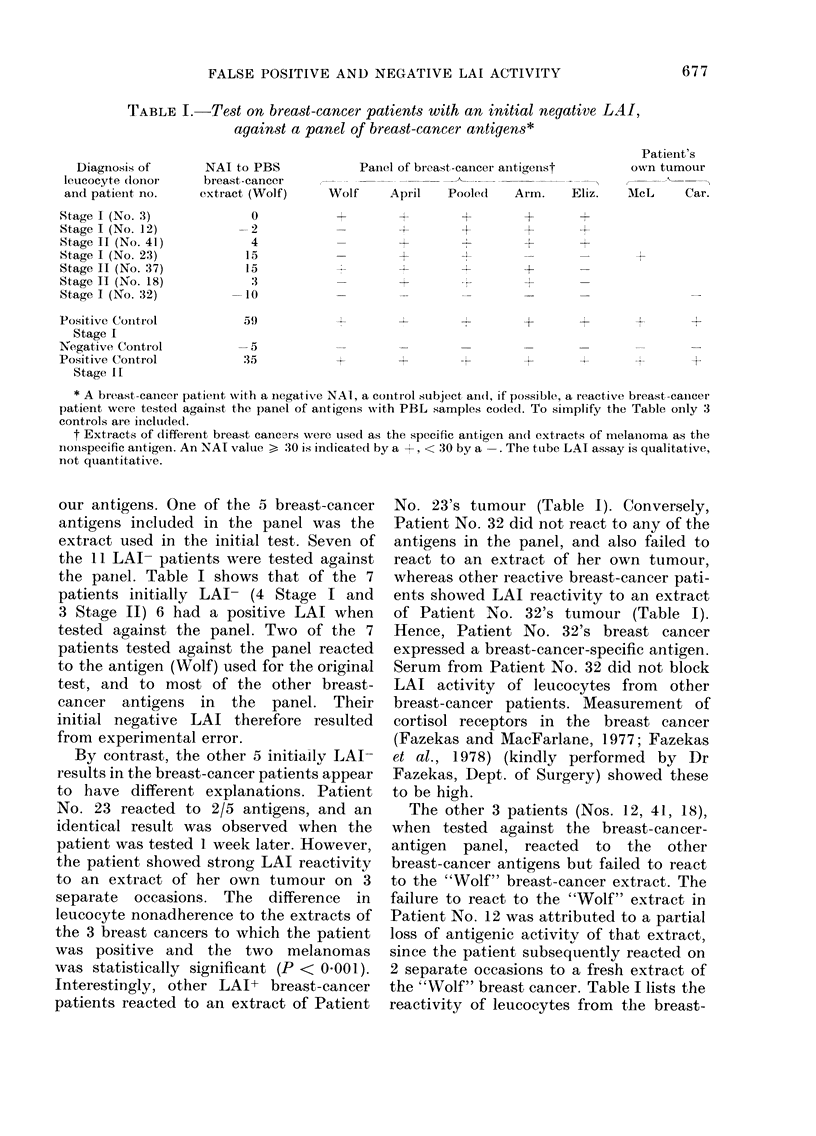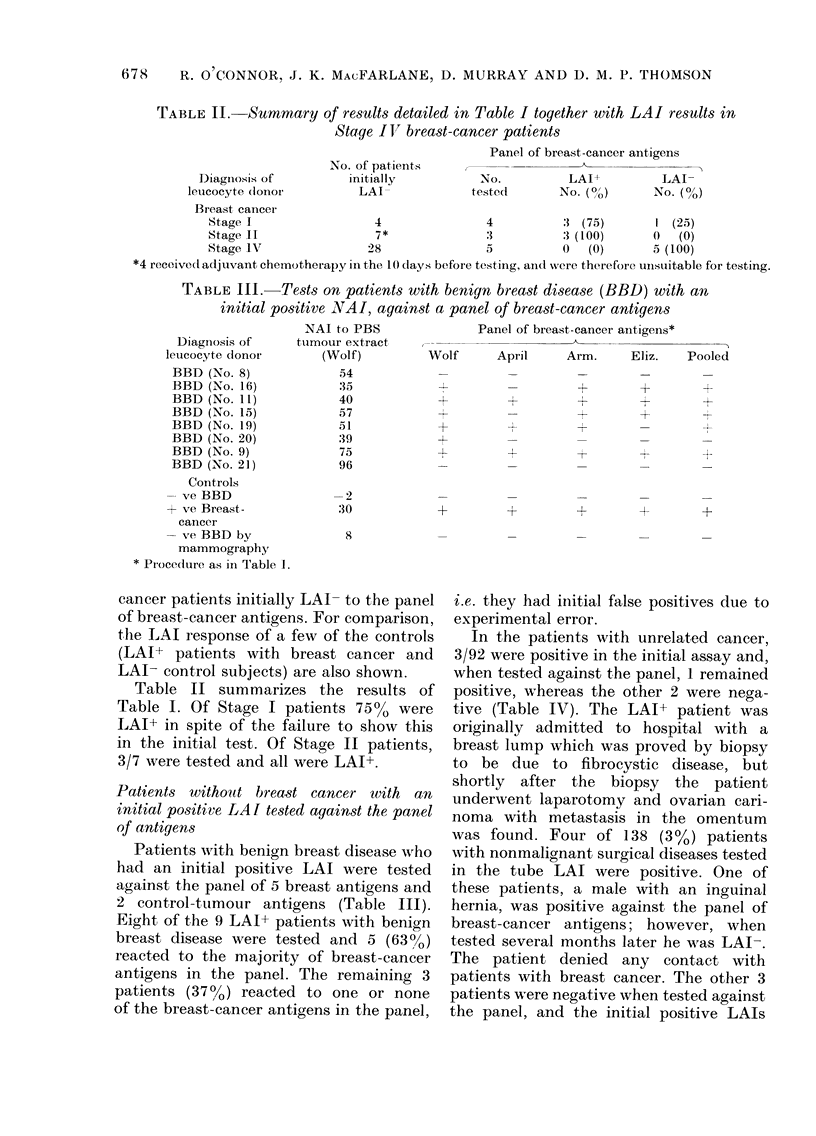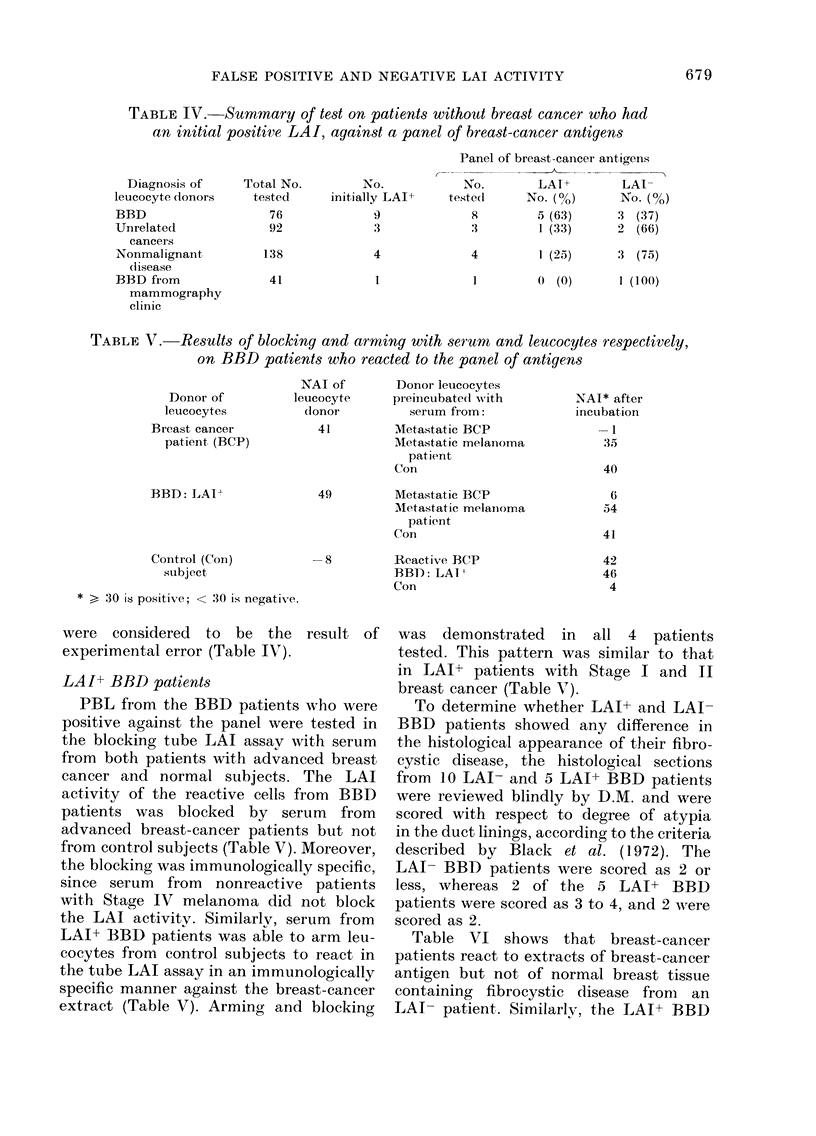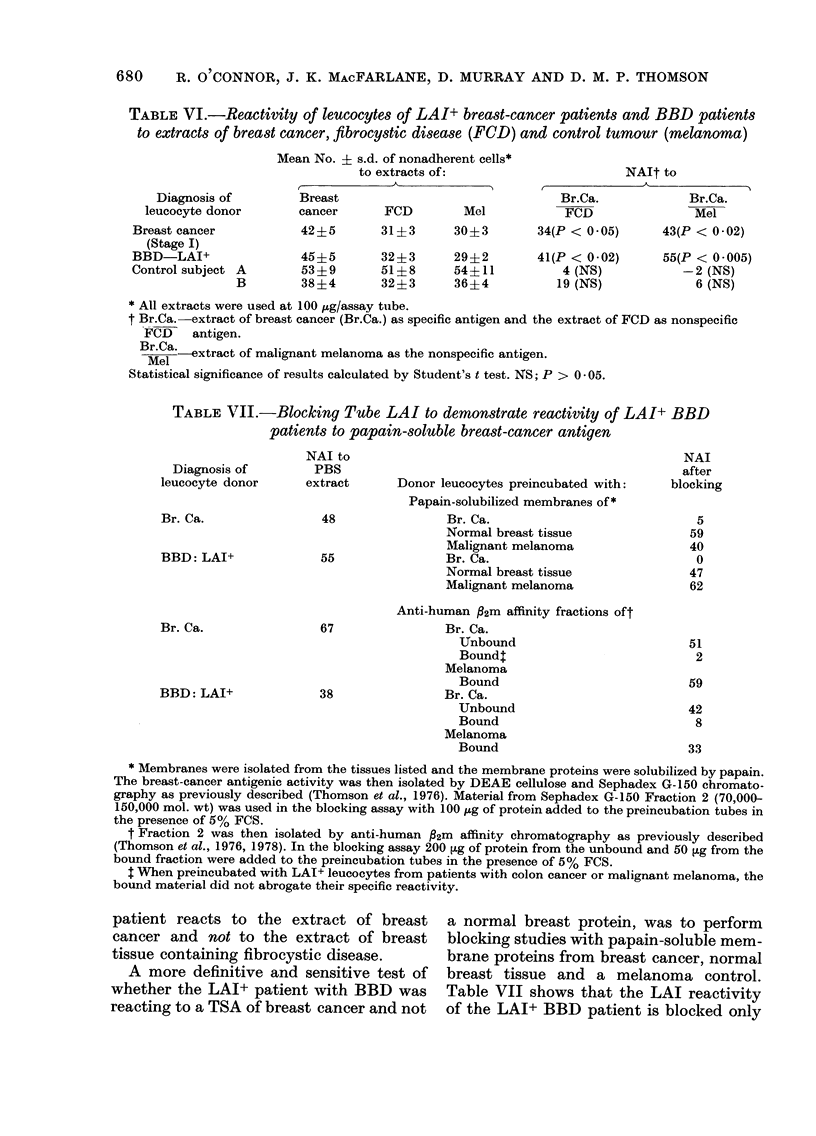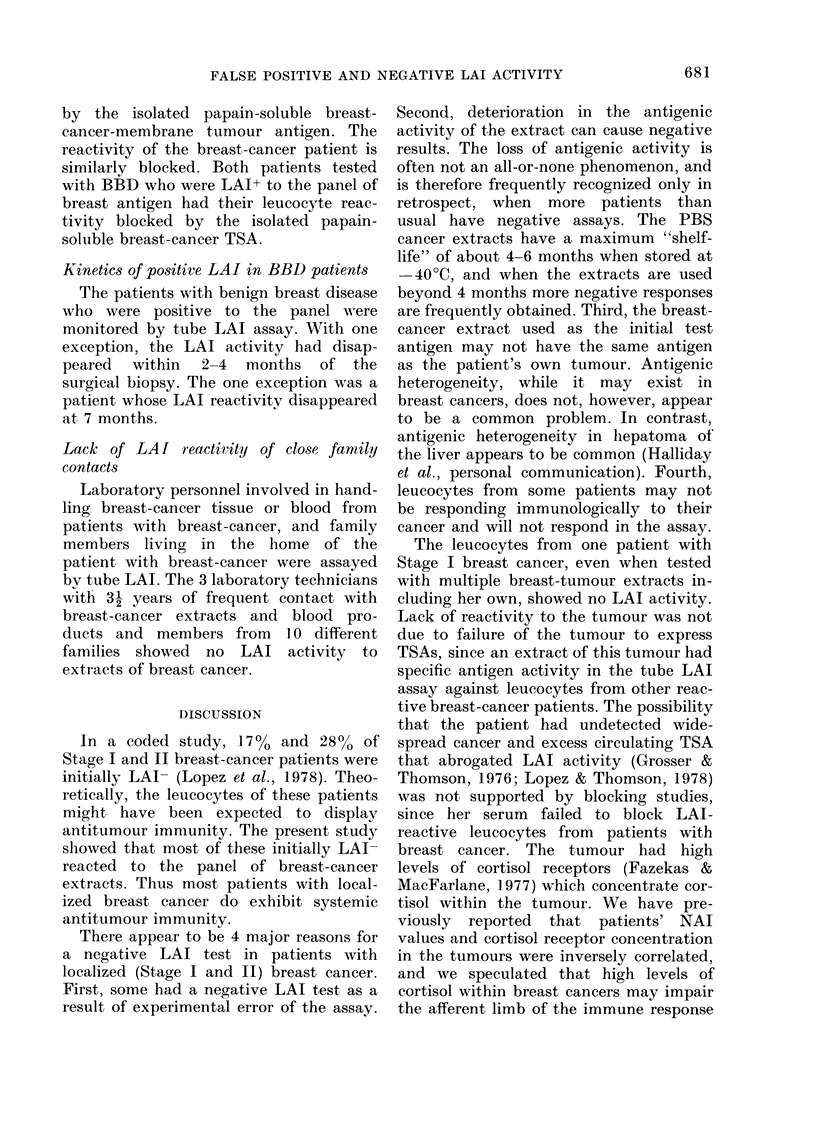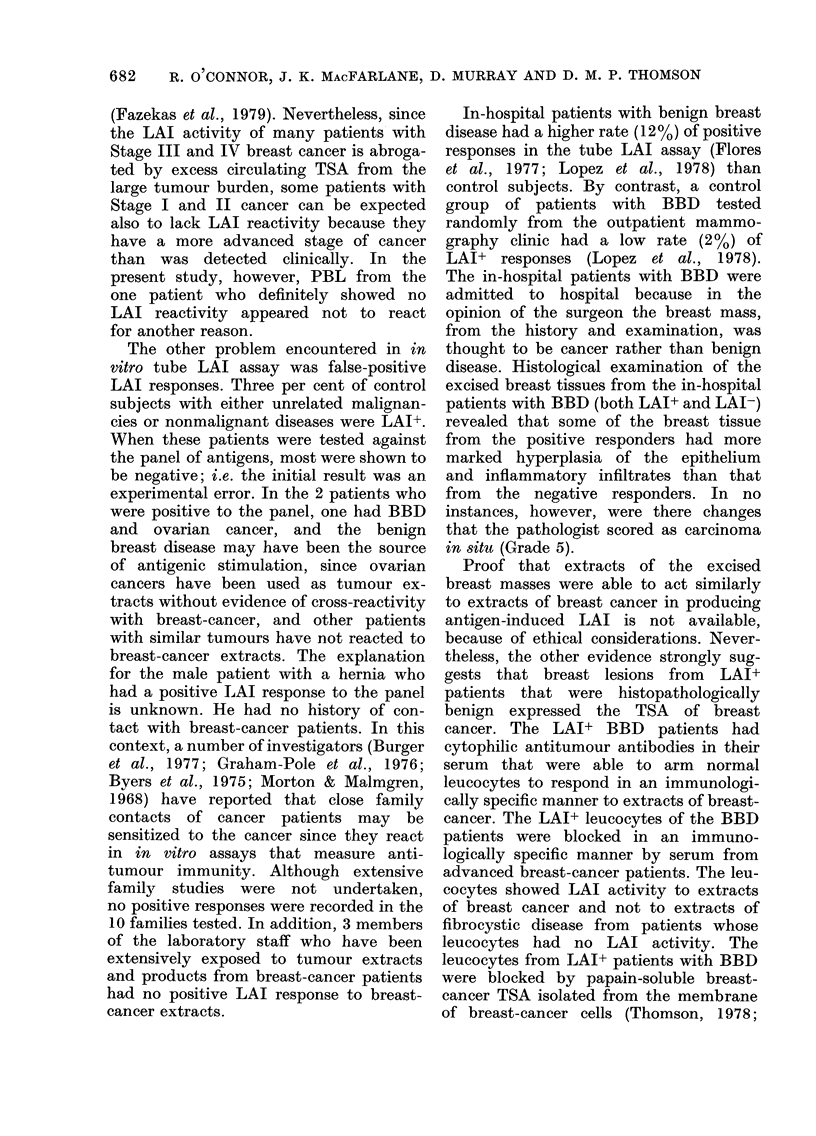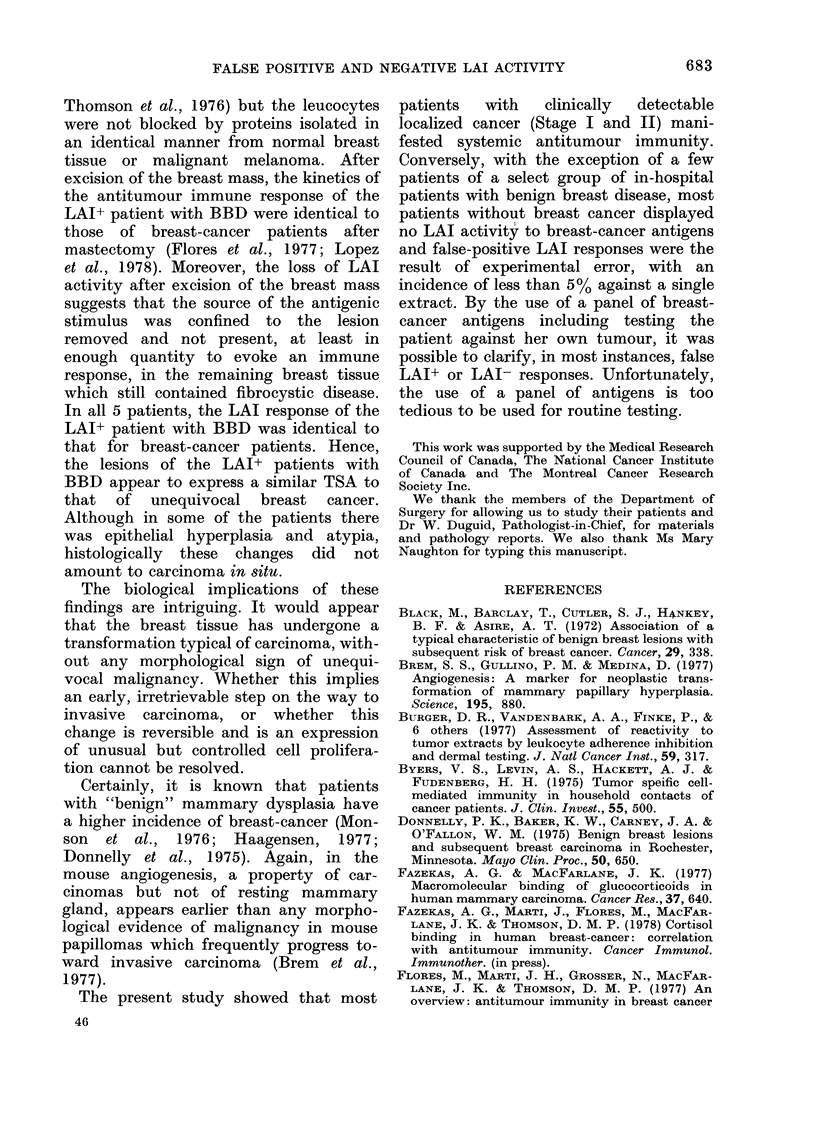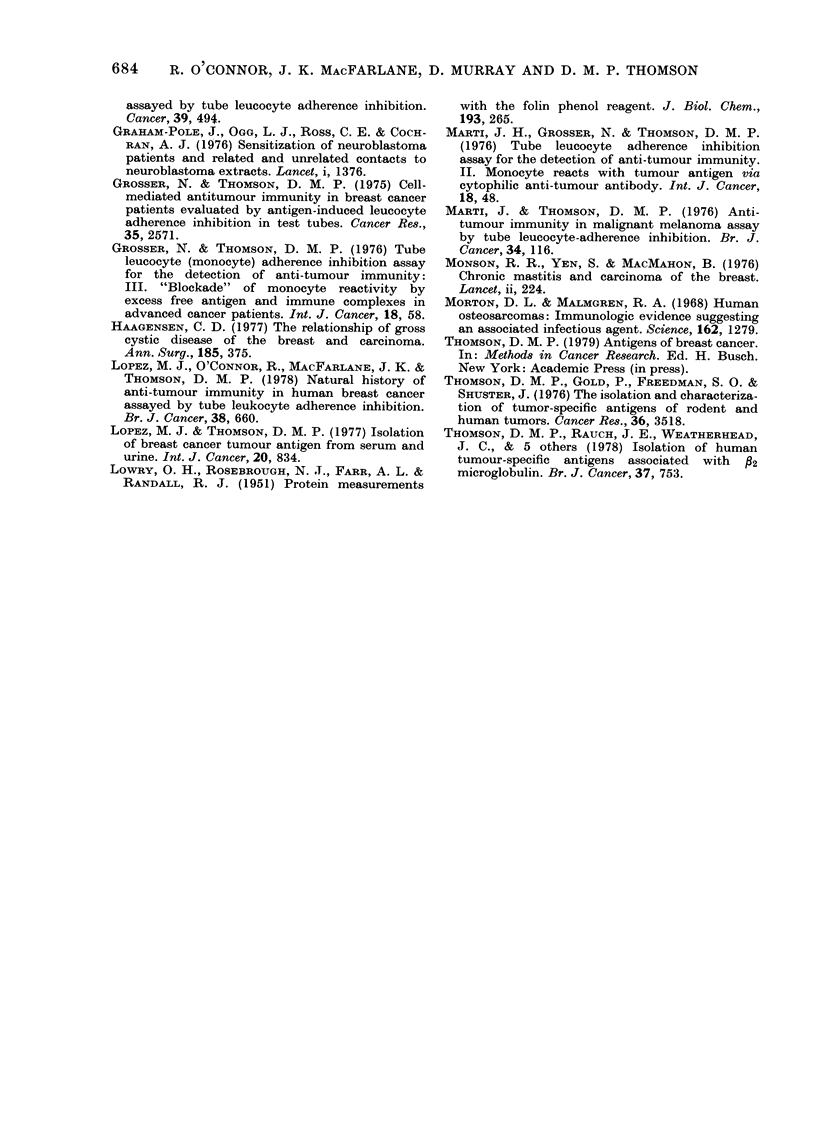# A study of false positive and negative responses in the tube leucocyte adherence inhibition (tube LAI) assay.

**DOI:** 10.1038/bjc.1978.272

**Published:** 1978-12

**Authors:** R. O'Connor, J. K. MacFarlane, D. Murray, D. M. Thomson

## Abstract

A panel of 5 different breast-cancer and 2 other cancer extracts was used to clarify the false-negative responses in patients with Stage I and II breast cancer and the false-positive responses in control subjects. Most patients with Stage I and II breast cancer who had an initially negative LAI response were positive when tested against the panel. The false negatives occurred because of (1) the experimental errors of the assay; (2) changes in the antigenic strength of the extracts; (3) antigenic heterogeneity of a few tumours and (4) lack of tumour-specific reactivity of the host. 3% of control subjects had a false-positive LAI response. The leucocytes from most of these positive patients did not react to the panel of antigens, and hence the false positives appeared to result from experimental error. In-hospital patients with benign breast disease had a 12% positivity rate when initially assayed, and 63% of these patients reacted to the panel of breast-cancer antigens. Those patients with benign breast disease who reacted to the panel of breast-cancer antigens had cytophilic anti-breast-cancer antibody in their serum; their leucocyte LAI reactivity was blocked in an immunologically specific manner by serum from advanced Stage IV breast-cancer patients; their leucocytes reacted to extracts of breast cancer and not fibrocystic breast tissue; their leucocyte reactivity was blocked by isolated breast-cancer TSA that was linked to beta 2 microglobulin, but not by normal breast-tissue proteins; and the kinetics of the LAI response after excision of the breast mass was identical to that observed with breast-cancer patients after mastectomy. In these patients, the breast tissue within the breast lump expressed breast TSA similar to unequivocal breast cancer.


*Br. J. Cancer* (1978) **38**, 674

### A STUDY OF FALSE POSITIVE AND NEGATIVE RESPONSES IN THE TUBE LEUCOCYTE ADHERENCE INHIBITION (TUBE LAI) ASSAY

R. O'CONNOR, J. K. MACFARLANE, D. MURRAY AND D. M. P. THOMSON*
(with the technical assistance of R. SCHWARTZ and J. WEATHERHEAD)

*From the Montreal General Hospital Research Institute, Division of Clinical Immunology and Department of Surgery, Montreal General Hospital, McGill University, Montreal, Canada H3G 1A4*

Received 5 June 1978 Accepted 4 September 1978

**Summary.—A panel of 5 different breast-cancer and 2 other cancer extracts was used to clarify the false-negative responses in patients with Stage I and II breast cancer and the false-positive responses in control subjects. Most patients with Stage I and II breast cancer who had an initially negative LAI response were positive when tested against the panel. The false negatives occurred because of (1) the experimental errors of the assay; (2) changes in the antigenic strength of the extracts; (3) antigenic heterogeneity of a few tumours and (4) lack of tumour-specific reactivity of the host. 3% of control subjects had a false-positive LAI response. The leucocytes from most of these positive patients did not react to the panel of antigens, and hence the false positives appeared to result from experimental error. In-hospital patients with benign breast disease had a 12% positivity rate when initially assayed, and 63% of these patients reacted to the panel of breast-cancer antigens. Those patients with benign breast disease who reacted to the panel of breast-cancer antigens had cytophilic anti-breast-cancer antibody in their serum; their leucocyte LAI reactivity was blocked in an immunologically specific manner by serum from advanced Stage IV breast-cancer patients; their leucocytes reacted to extracts of breast cancer and not fibrocystic breast tissue; their leucocyte reactivity was blocked by isolated breast-cancer TSA that was linked to $\beta_2$ microglobulin, but not by normal breast-tissue proteins; and the kinetics of the LAI response after excision of the breast mass was identical to that observed with breast-cancer patients after mastectomy. In these patients, the breast tissue within the breast lump expressed breast TSA similar to unequivocal breast cancer.**

LEUCOCYTES from about 80% of patients with Stage I and II breast cancer reacted in the tube LAI assay (Grosser & Thomson, 1975; Flores *et al.*, 1977; Lopez *et al.*, 1978) whereas leucocytes from less than 5% of control subjects reacted in the assay. Although the latter figure was considered to result from the variability of a biological assay, no systematic study of the false-positive patients had been undertaken to prove that the patients were not, in fact, sensitized to a breast-cancer antigen. Conversely, there was no explanation why a minority of patients with Stage I and II breast cancer did not react in the tube LAI assay. In addition, patients with benign breast disease had a higher rate of LAI positivity (Lopez *et al.*, 1978; Flores *et al.*, 1977) than other control subjects, and this raised the possibility that some of these patients were reacting to breast-cancer antigens (Flores *et al.*, 1977; Lopez *et al.*, 1978).

Because the tube LAI is a reliable assay, reproducible *in vitro*, for the

* To whom reprint requests should be addressed, at The Montreal General Hospital, 1650 Cedar Avenue, Montreal, Canada H3G 1A4.

detection of sensitization to tumour-specific antigens (Grosser & Thomson, 1975; Marti & Thomson, 1976; Flores *et al.*, 1977; Lopez *et al.*, 1978; Thomson, 1978) these observations were considered to be accurate, and the present study was undertaken to define, if possible, the reason for the false-positive and negative LAI responses. To investigate these, the leucocytes from the subject were reacted against a panel of extracts of breast cancer and unrelated tumour. The panel of antigens was used on the assumption that the leucocytes from a false-negative patient might show reactivity to other breast-cancer extracts if there was some heterogeneity of breast-cancer tumour antigens, or if the initial test antigen had lost its activity. On the other hand, the panel of antigens was used to test leucocytes from patients who had false-positive reactions, on the assumption that if the leucocytes recognized a tumour-specific antigen, they should react to most other extracts of breast cancer. If the positive response to the initial test antigen resulted from some idiosyncrasy of the initial test extract, or from experimental error, an LAI response to different breast-cancer extracts would not be expected. The validity of this approach for clarifying the false-positive and negative LAI responses is shown in the results presented in this study.

### MATERIALS AND METHODS

*Subjects.*—The patients with false-positive and negative LAI responses were drawn from a total of 451 patients tested in the coded study of Lopez *et al.* (1978).

*Tumour extracts and tumour panels.*—The preparation of the cancer extracts has been previously described in detail (Grosser & Thomson, 1975; Marti & Thomson, 1976; Flores *et al.*, 1977; Lopez *et al.*, 1978; Thomson, 1978). Four breast-cancer extracts were prepared from metastatic deposits in the liver from different postmortem specimens. An extract was also prepared from 5 primary breast cancers that were pooled. In 2 instances, an extract was prepared from the patient's own breast cancer. The nonspecific antigens were prepared from metastatic malignant melanoma and fibrocystic disease of the breast. The breast-cancer and melanoma extracts were the panel against which patients who had false-positive or false-negative LAI responses were tested.

When a patient was tested against the panel of antigens, a control subject and, if possible, a breast-cancer patient whose leucocytes were $LAI^+$ were tested at the same time. In addition, control subjects and breast-cancer patients whose leucocytes were $LAI^+$ were tested with the panel of extracts, to show that the leucocytes from the breast-cancer patients reacted to each of the breast-cancer antigens used in the panel, and that the leucocytes from control subjects did not react. The leucocytes to be tested were coded and the code was broken at the completion of the assay.

Protein concentration was determined by the method of Lowry *et al.* (1951) with bovine albumin as a standard. The protein concentration of the specific and nonspecific extracts was $100 \pm 10\ \mu g$ per assay tube, since this amount was optimal (Grosser & Thomson, 1975; Marti & Thomson, 1976; Flores *et al.*, 1977; Lopez *et al.*, 1978; Thomson, 1978). Each sample of tumour extract for the panel was used once and then discarded; samples were stored at −40°C.

*Antigen-induced tube leucocyte-adherence inhibition (tube LAI) assay.*—In a series of test tubes, $10^6$ peripheral-blood leucocytes (PBL) were plated in the presence of extracts of specific breast cancer, control melanoma and Medium 199 as previously described (Grosser & Thomson, 1975; Marti & Thomson, 1976; Flores *et al.*, 1977; Lopez *et al.*, 1978; Thomson, 1978). The tubes were incubated horizontally at 37°C in a 5% $CO_2$ humidified atmosphere and after 2 h the number of nonadherent cells was counted in a haemocytometer and the nonadherence index (NAI) was calculated as previously described (Grosser & Thomson, 1975; & others).

An NAI value $\geqslant 30$ was positive, $< 30$ was negative (Flores *et al.*, 1977; Lopez *et al.*, 1978; Thomson, 1978).

*Arming of control leucocytes by serum from reactive patients.*—PBL from control subjects were preincubated with serum from reactive breast-cancer patients, control subjects and from the patient with benign breast disease whose leucocytes showed LAI reactivity. After preincubation with the serum, the PBL were freed of excess serum by washing twice with 10 ml of Medium 199, and then plated in the standard antigen-induced tube LAI assay (Marti *et al.*, 1976). The non-adherent cells were counted after 2 h incubation and the NAI calculated.

*Blocking of LAI activiry by serum and papain-soluble antigens.*—PBL from reactive breast-cancer patients (Stages I and II), from control subjects with negative LAI assays, and from patients with benign breast disease with a positive LAI assay, were preincubated with serum from either advanced nonreactive Stage IV breast-cancer patients or control subjects. After incubation for 30 min at 37°C in a 5% $CO_3$ atmosphere, the cells were washed with 10 ml of Medium 199 to remove excess serum, and the PBL were then plated in the standard tube LAI assay (Thomson, 1978; Grosser & Thomson, 1976). After 2h incubation the nonadherent cells were counted and the NAI calculated. Papain-soluble material from the membranes of breast-cancer, normal breast tissue and melanoma were similarly tested for blocking activity (Thomson, 1978; Thomson *et al.*, 1978).

### RESULTS

Table II of the paper by Lopez *et al.* (1978) shows the results of a coded study of the tube LAI assay in breast-cancer patients and a variety of control subjects. In Stage I and II breast cancer, 4/24 (17%) and 7/26 (28%) patients respectively did not have leucocyte reactivity in the tube LAI assay. In the group of patients admitted to hospital with breast masses which clinically were thought to be breast cancer but turned out to be benign breast disease (BBD), 9/76 (12%) had positive LAI, whereas in the group of outpatients with benign breast disease 1/41 (2%) had positive LAI. To date, no breast cancers have developed in either group of BBD patients. Control subjects with benign surgical disease and unrelated cancer had 3/92 (3%) and 4/138 (3%) respectively with LAI reactivity.

To determine whether a panel of breast-cancer extracts could be used to test breast-cancer patients with limited cancer who had a negative LAI response, and patients without breast cancer who had a positive LAI response, 5 breast-cancer patients who were $LAI^+$ and 5 control subjects who were $LAI^-$ had their leucocytes tested against the panel. All samples of leucocytes were coded. Since the PBL were tested against 5 breast-cancer and 2 melanoma antigens, the combination resulted in 10 tests to the breast cancers. The leucocytes from 5 breast-cancer patients showed positive LAI activity to the 5 different breast-cancer antigens in the panel, except for one patient with a negative response to one antigen. The leucocytes from the 5 control subjects had a positive response in one of the tests to the breast cancer antigens, which was a 4% rate of experimental error. The results suggested that the experimental error was low enough for PBL responses to the panel of breast-cancer extracts to be evaluated.

Throughout the year, leucocytes from reactive breast-cancer patients and control subjects were repeatedly tested against the panel of antigens to continue to assess the reproducibility of the panel. If leucocytes from patients with limited malignant melanoma were incubated with the extracts of malignant melanoma (non-specific) and breast cancer (specific), the melanoma extracts produced specific antigen-induced LAI. Classical criss-cross experiments were not done routinely, because it was virtually impossible to have available at the same time patients with breast cancer and melanoma who were $LAI^+$ to their respective tumours.

*Breast-cancer patients (Stages I and II) with an initial negative LAI, tested against the panel of antigens*

To investigate why some patients with localized cancer (Stage I and II) had a negative LAI before surgery, these patients were tested 3–4 weeks after surgery, against a panel of 5 different breast-cancer antigens and two nonspecific tum-

TABLE I.—*Test on breast-cancer patients with an initial negative LAI, against a panel of breast-cancer antigens**

| Diagnosis of leucocyte donor and patient no. | NAI to PBS breast-cancer extract (Wolf) | Panel of breast-cancer antigens† | | | | | Patient's own tumour | |
|---|---|---|---|---|---|---|---|---|
| | | Wolf | April | Pooled | Arm. | Eliz. | McL | Car. |
| Stage I (No. 3) | 0 | + | + | + | + | + | | |
| Stage I (No. 12) | −2 | − | + | + | + | + | | |
| Stage II (No. 41) | 4 | − | + | + | + | + | | |
| Stage I (No. 23) | 15 | − | + | + | − | − | + | |
| Stage II (No. 37) | 15 | + | + | + | + | − | | |
| Stage II (No. 18) | 3 | − | + | + | + | − | | |
| Stage I (No. 32) | −10 | − | − | − | − | − | | − |
| Positive Control Stage I | 59 | + | + | + | + | + | + | + |
| Negative Control | −5 | − | − | − | − | − | − | − |
| Positive Control Stage II | 35 | + | + | + | + | + | + | + |

* A breast-cancer patient with a negative NAI, a control subject and, if possible, a reactive breast-cancer patient were tested against the panel of antigens with PBL samples coded. To simplify the Table only 3 controls are included.

† Extracts of different breast cancers were used as the specific antigen and extracts of melanoma as the nonspecific antigen. An NAI value ⩾ 30 is indicated by a +, < 30 by a −. The tube LAI assay is qualitative, not quantitative.

our antigens. One of the 5 breast-cancer antigens included in the panel was the extract used in the initial test. Seven of the 11 LAI$^-$ patients were tested against the panel. Table I shows that of the 7 patients initially LAI$^-$ (4 Stage I and 3 Stage II) 6 had a positive LAI when tested against the panel. Two of the 7 patients tested against the panel reacted to the antigen (Wolf) used for the original test, and to most of the other breast-cancer antigens in the panel. Their initial negative LAI therefore resulted from experimental error.

By contrast, the other 5 initially LAI$^-$ results in the breast-cancer patients appear to have different explanations. Patient No. 23 reacted to 2/5 antigens, and an identical result was observed when the patient was tested 1 week later. However, the patient showed strong LAI reactivity to an extract of her own tumour on 3 separate occasions. The difference in leucocyte nonadherence to the extracts of the 3 breast cancers to which the patient was positive and the two melanomas was statistically significant ($P < 0{\cdot}001$). Interestingly, other LAI$^+$ breast-cancer patients reacted to an extract of Patient No. 23's tumour (Table I). Conversely, Patient No. 32 did not react to any of the antigens in the panel, and also failed to react to an extract of her own tumour, whereas other reactive breast-cancer patients showed LAI reactivity to an extract of Patient No. 32's tumour (Table I). Hence, Patient No. 32's breast cancer expressed a breast-cancer-specific antigen. Serum from Patient No. 32 did not block LAI activity of leucocytes from other breast-cancer patients. Measurement of cortisol receptors in the breast cancer (Fazekas and MacFarlane, 1977; Fazekas *et al.*, 1978) (kindly performed by Dr Fazekas, Dept. of Surgery) showed these to be high.

The other 3 patients (Nos. 12, 41, 18), when tested against the breast-cancer-antigen panel, reacted to the other breast-cancer antigens but failed to react to the "Wolf" breast-cancer extract. The failure to react to the "Wolf" extract in Patient No. 12 was attributed to a partial loss of antigenic activity of that extract, since the patient subsequently reacted on 2 separate occasions to a fresh extract of the "Wolf" breast cancer. Table I lists the reactivity of leucocytes from the breast-

TABLE II.—*Summary of results detailed in Table I together with LAI results in Stage IV breast-cancer patients*

| Diagnosis of leucocyte donor | No. of patients initially LAI⁻ | Panel of breast-cancer antigens No. tested | LAI+ No. (%) | LAI− No. (%) |
|---|---|---|---|---|
| Breast cancer | | | | |
| Stage I | 4 | 4 | 3 (75) | 1 (25) |
| Stage II | 7* | 3 | 3 (100) | 0 (0) |
| Stage IV | 28 | 5 | 0 (0) | 5 (100) |

*4 received adjuvant chemotherapy in the 10 days before testing, and were therefore unsuitable for testing.

TABLE III.—*Tests on patients with benign breast disease (BBD) with an initial positive NAI, against a panel of breast-cancer antigens*

| Diagnosis of leucocyte donor | NAI to PBS tumour extract (Wolf) | Panel of breast-cancer antigens* Wolf | April | Arm. | Eliz. | Pooled |
|---|---|---|---|---|---|---|
| BBD (No. 8) | 54 | − | − | − | − | − |
| BBD (No. 16) | 35 | + | − | + | + | + |
| BBD (No. 11) | 40 | + | + | + | + | + |
| BBD (No. 15) | 57 | + | − | + | + | + |
| BBD (No. 19) | 51 | + | + | + | − | + |
| BBD (No. 20) | 39 | + | − | − | − | − |
| BBD (No. 9) | 75 | + | + | + | + | + |
| BBD (No. 21) | 96 | − | − | − | − | − |
| Controls | | | | | | |
| − ve BBD | −2 | − | − | − | − | − |
| + ve Breast-cancer | 30 | + | + | + | + | + |
| − ve BBD by mammography | 8 | − | − | − | − | − |

* Procedure as in Table I.

cancer patients initially LAI⁻ to the panel of breast-cancer antigens. For comparison, the LAI response of a few of the controls (LAI+ patients with breast cancer and LAI⁻ control subjects) are also shown.

Table II summarizes the results of Table I. Of Stage I patients 75% were LAI+ in spite of the failure to show this in the initial test. Of Stage II patients, 3/7 were tested and all were LAI+.

*Patients without breast cancer with an initial positive LAI tested against the panel of antigens*

Patients with benign breast disease who had an initial positive LAI were tested against the panel of 5 breast antigens and 2 control-tumour antigens (Table III). Eight of the 9 LAI+ patients with benign breast disease were tested and 5 (63%) reacted to the majority of breast-cancer antigens in the panel. The remaining 3 patients (37%) reacted to one or none of the breast-cancer antigens in the panel, *i.e.* they had initial false positives due to experimental error.

In the patients with unrelated cancer, 3/92 were positive in the initial assay and, when tested against the panel, 1 remained positive, whereas the other 2 were negative (Table IV). The LAI+ patient was originally admitted to hospital with a breast lump which was proved by biopsy to be due to fibrocystic disease, but shortly after the biopsy the patient underwent laparotomy and ovarian carinoma with metastasis in the omentum was found. Four of 138 (3%) patients with nonmalignant surgical diseases tested in the tube LAI were positive. One of these patients, a male with an inguinal hernia, was positive against the panel of breast-cancer antigens; however, when tested several months later he was LAI⁻. The patient denied any contact with patients with breast cancer. The other 3 patients were negative when tested against the panel, and the initial positive LAIs

TABLE IV.—*Summary of test on patients without breast cancer who had an initial positive LAI, against a panel of breast-cancer antigens*

| | | | Panel of breast-cancer antigens | | |
|---|---|---|---|---|---|
| Diagnosis of leucocyte donors | Total No. tested | No. initially LAI+ | No. tested | LAI+ No. (%) | LAI− No. (%) |
| BBD | 76 | 9 | 8 | 5 (63) | 3 (37) |
| Unrelated cancers | 92 | 3 | 3 | 1 (33) | 2 (66) |
| Nonmalignant disease | 138 | 4 | 4 | 1 (25) | 3 (75) |
| BBD from mammography clinic | 41 | 1 | 1 | 0 (0) | 1 (100) |

TABLE V.—*Results of blocking and arming with serum and leucocytes respectively, on BBD patients who reacted to the panel of antigens*

| Donor of leucocytes | NAI of leucocyte donor | Donor leucocytes preincubated with serum from: | NAI* after incubation |
|---|---|---|---|
| Breast cancer patient (BCP) | 41 | Metastatic BCP | −1 |
| | | Metastatic melanoma patient | 35 |
| | | Con | 40 |
| BBD: LAI+ | 49 | Metastatic BCP | 6 |
| | | Metastatic melanoma patient | 54 |
| | | Con | 41 |
| Control (Con) subject | −8 | Reactive BCP | 42 |
| | | BBD: LAI+ | 46 |
| | | Con | 4 |

* ⩾ 30 is positive; < 30 is negative.

were considered to be the result of experimental error (Table IV).

*LAI+ BBD patients*

PBL from the BBD patients who were positive against the panel were tested in the blocking tube LAI assay with serum from both patients with advanced breast cancer and normal subjects. The LAI activity of the reactive cells from BBD patients was blocked by serum from advanced breast-cancer patients but not from control subjects (Table V). Moreover, the blocking was immunologically specific, since serum from nonreactive patients with Stage IV melanoma did not block the LAI activity. Similarly, serum from LAI+ BBD patients was able to arm leucocytes from control subjects to react in the tube LAI assay in an immunologically specific manner against the breast-cancer extract (Table V). Arming and blocking was demonstrated in all 4 patients tested. This pattern was similar to that in LAI+ patients with Stage I and II breast cancer (Table V).

To determine whether LAI+ and LAI− BBD patients showed any difference in the histological appearance of their fibrocystic disease, the histological sections from 10 LAI− and 5 LAI+ BBD patients were reviewed blindly by D.M. and were scored with respect to degree of atypia in the duct linings, according to the criteria described by Black *et al.* (1972). The LAI− BBD patients were scored as 2 or less, whereas 2 of the 5 LAI+ BBD patients were scored as 3 to 4, and 2 were scored as 2.

Table VI shows that breast-cancer patients react to extracts of breast-cancer antigen but not of normal breast tissue containing fibrocystic disease from an LAI− patient. Similarly, the LAI+ BBD

TABLE VI.—*Reactivity of leucocytes of LAI+ breast-cancer patients and BBD patients to extracts of breast cancer, fibrocystic disease (FCD) and control tumour (melanoma)*

| Diagnosis of leucocyte donor | Mean No. ± s.d. of nonadherent cells* to extracts of: Breast cancer | FCD | Mel | NAI† to: $\frac{\text{Br.Ca.}}{\text{FCD}}$ | $\frac{\text{Br.Ca.}}{\text{Mel}}$ |
|---|---|---|---|---|---|
| Breast cancer (Stage I) | 42±5 | 31±3 | 30±3 | 34($P < 0{\cdot}05$) | 43($P < 0{\cdot}02$) |
| BBD—LAI+ | 45±5 | 32±3 | 29±2 | 41($P < 0{\cdot}02$) | 55($P < 0{\cdot}005$) |
| Control subject A | 53±9 | 51±8 | 54±11 | 4 (NS) | −2 (NS) |
| B | 38±4 | 32±3 | 36±4 | 19 (NS) | 6 (NS) |

* All extracts were used at 100 μg/assay tube.

† $\frac{\text{Br.Ca.}}{\text{FCD}}$—extract of breast cancer (Br.Ca.) as specific antigen and the extract of FCD as nonspecific antigen.

$\frac{\text{Br.Ca.}}{\text{Mel}}$—extract of malignant melanoma as the nonspecific antigen.

Statistical significance of results calculated by Student's $t$ test. NS; $P > 0{\cdot}05$.

TABLE VII.—*Blocking Tube LAI to demonstrate reactivity of LAI+ BBD patients to papain-soluble breast-cancer antigen*

| Diagnosis of leucocyte donor | NAI to PBS extract | Donor leucocytes preincubated with: | NAI after blocking |
|---|---|---|---|
| | | Papain-solubilized membranes of* | |
| Br. Ca. | 48 | Br. Ca. | 5 |
| | | Normal breast tissue | 59 |
| | | Malignant melanoma | 40 |
| BBD: LAI+ | 55 | Br. Ca. | 0 |
| | | Normal breast tissue | 47 |
| | | Malignant melanoma | 62 |
| | | Anti-human $\beta_2$m affinity fractions of† | |
| Br. Ca. | 67 | Br. Ca. | |
| | | Unbound | 51 |
| | | Bound‡ | 2 |
| | | Melanoma | |
| | | Bound | 59 |
| BBD: LAI+ | 38 | Br. Ca. | |
| | | Unbound | 42 |
| | | Bound | 8 |
| | | Melanoma | |
| | | Bound | 33 |

* Membranes were isolated from the tissues listed and the membrane proteins were solubilized by papain. The breast-cancer antigenic activity was then isolated by DEAE cellulose and Sephadex G-150 chromatography as previously described (Thomson *et al.*, 1976). Material from Sephadex G-150 Fraction 2 (70,000–150,000 mol. wt) was used in the blocking assay with 100 μg of protein added to the preincubation tubes in the presence of 5% FCS.

† Fraction 2 was then isolated by anti-human $\beta_2$m affinity chromatography as previously described (Thomson *et al.*, 1976, 1978). In the blocking assay 200 μg of protein from the unbound and 50 μg from the bound fraction were added to the preincubation tubes in the presence of 5% FCS.

‡ When preincubated with LAI+ leucocytes from patients with colon cancer or malignant melanoma, the bound material did not abrogate their specific reactivity.

patient reacts to the extract of breast cancer and *not* to the extract of breast tissue containing fibrocystic disease.

A more definitive and sensitive test of whether the LAI+ patient with BBD was reacting to a TSA of breast cancer and not a normal breast protein, was to perform blocking studies with papain-soluble membrane proteins from breast cancer, normal breast tissue and a melanoma control. Table VII shows that the LAI reactivity of the LAI+ BBD patient is blocked only by the isolated papain-soluble breast-cancer-membrane tumour antigen. The reactivity of the breast-cancer patient is similarly blocked. Both patients tested with BBD who were $LAI^+$ to the panel of breast antigen had their leucocyte reactivity blocked by the isolated papain-soluble breast-cancer TSA.

*Kinetics of positive LAI in BBD patients*

The patients with benign breast disease who were positive to the panel were monitored by tube LAI assay. With one exception, the LAI activity had disappeared within 2–4 months of the surgical biopsy. The one exception was a patient whose LAI reactivity disappeared at 7 months.

*Lack of LAI reactivity of close family contacts*

Laboratory personnel involved in handling breast-cancer tissue or blood from patients with breast-cancer, and family members living in the home of the patient with breast-cancer were assayed by tube LAI. The 3 laboratory technicians with 3½ years of frequent contact with breast-cancer extracts and blood products and members from 10 different families showed no LAI activity to extracts of breast cancer.

### DISCUSSION

In a coded study, 17% and 28% of Stage I and II breast-cancer patients were initially $LAI^-$ (Lopez *et al.*, 1978). Theoretically, the leucocytes of these patients might have been expected to display antitumour immunity. The present study showed that most of these initially $LAI^-$ reacted to the panel of breast-cancer extracts. Thus most patients with localized breast cancer do exhibit systemic antitumour immunity.

There appear to be 4 major reasons for a negative LAI test in patients with localized (Stage I and II) breast cancer. First, some had a negative LAI test as a result of experimental error of the assay. Second, deterioration in the antigenic activity of the extract can cause negative results. The loss of antigenic activity is often not an all-or-none phenomenon, and is therefore frequently recognized only in retrospect, when more patients than usual have negative assays. The PBS cancer extracts have a maximum "shelf-life" of about 4–6 months when stored at −40°C, and when the extracts are used beyond 4 months more negative responses are frequently obtained. Third, the breast-cancer extract used as the initial test antigen may not have the same antigen as the patient's own tumour. Antigenic heterogeneity, while it may exist in breast cancers, does not, however, appear to be a common problem. In contrast, antigenic heterogeneity in hepatoma of the liver appears to be common (Halliday *et al.*, personal communication). Fourth, leucocytes from some patients may not be responding immunologically to their cancer and will not respond in the assay.

The leucocytes from one patient with Stage I breast cancer, even when tested with multiple breast-tumour extracts including her own, showed no LAI activity. Lack of reactivity to the tumour was not due to failure of the tumour to express TSAs, since an extract of this tumour had specific antigen activity in the tube LAI assay against leucocytes from other reactive breast-cancer patients. The possibility that the patient had undetected widespread cancer and excess circulating TSA that abrogated LAI activity (Grosser & Thomson, 1976; Lopez & Thomson, 1978) was not supported by blocking studies, since her serum failed to block LAI-reactive leucocytes from patients with breast cancer. The tumour had high levels of cortisol receptors (Fazekas & MacFarlane, 1977) which concentrate cortisol within the tumour. We have previously reported that patients' NAI values and cortisol receptor concentration in the tumours were inversely correlated, and we speculated that high levels of cortisol within breast cancers may impair the afferent limb of the immune response

(Fazekas *et al.*, 1979). Nevertheless, since the LAI activity of many patients with Stage III and IV breast cancer is abrogated by excess circulating TSA from the large tumour burden, some patients with Stage I and II cancer can be expected also to lack LAI reactivity because they have a more advanced stage of cancer than was detected clinically. In the present study, however, PBL from the one patient who definitely showed no LAI reactivity appeared not to react for another reason.

The other problem encountered in *in vitro* tube LAI assay was false-positive LAI responses. Three per cent of control subjects with either unrelated malignancies or nonmalignant diseases were $LAI^+$. When these patients were tested against the panel of antigens, most were shown to be negative; *i.e.* the initial result was an experimental error. In the 2 patients who were positive to the panel, one had BBD and ovarian cancer, and the benign breast disease may have been the source of antigenic stimulation, since ovarian cancers have been used as tumour extracts without evidence of cross-reactivity with breast-cancer, and other patients with similar tumours have not reacted to breast-cancer extracts. The explanation for the male patient with a hernia who had a positive LAI response to the panel is unknown. He had no history of contact with breast-cancer patients. In this context, a number of investigators (Burger *et al.*, 1977; Graham-Pole *et al.*, 1976; Byers *et al.*, 1975; Morton & Malmgren, 1968) have reported that close family contacts of cancer patients may be sensitized to the cancer since they react in *in vitro* assays that measure antitumour immunity. Although extensive family studies were not undertaken, no positive responses were recorded in the 10 families tested. In addition, 3 members of the laboratory staff who have been extensively exposed to tumour extracts and products from breast-cancer patients had no positive LAI response to breast-cancer extracts.

In-hospital patients with benign breast disease had a higher rate (12%) of positive responses in the tube LAI assay (Flores *et al.*, 1977; Lopez *et al.*, 1978) than control subjects. By contrast, a control group of patients with BBD tested randomly from the outpatient mammography clinic had a low rate (2%) of $LAI^+$ responses (Lopez *et al.*, 1978). The in-hospital patients with BBD were admitted to hospital because in the opinion of the surgeon the breast mass, from the history and examination, was thought to be cancer rather than benign disease. Histological examination of the excised breast tissues from the in-hospital patients with BBD (both $LAI^+$ and $LAI^-$) revealed that some of the breast tissue from the positive responders had more marked hyperplasia of the epithelium and inflammatory infiltrates than that from the negative responders. In no instances, however, were there changes that the pathologist scored as carcinoma *in situ* (Grade 5).

Proof that extracts of the excised breast masses were able to act similarly to extracts of breast cancer in producing antigen-induced LAI is not available, because of ethical considerations. Nevertheless, the other evidence strongly suggests that breast lesions from $LAI^+$ patients that were histopathologically benign expressed the TSA of breast cancer. The $LAI^+$ BBD patients had cytophilic antitumour antibodies in their serum that were able to arm normal leucocytes to respond in an immunologically specific manner to extracts of breast-cancer. The $LAI^+$ leucocytes of the BBD patients were blocked in an immunologically specific manner by serum from advanced breast-cancer patients. The leucocytes showed LAI activity to extracts of breast cancer and not to extracts of fibrocystic disease from patients whose leucocytes had no LAI activity. The leucocytes from $LAI^+$ patients with BBD were blocked by papain-soluble breast-cancer TSA isolated from the membrane of breast-cancer cells (Thomson, 1978;

Thomson *et al.*, 1976) but the leucocytes were not blocked by proteins isolated in an identical manner from normal breast tissue or malignant melanoma. After excision of the breast mass, the kinetics of the antitumour immune response of the LAI+ patient with BBD were identical to those of breast-cancer patients after mastectomy (Flores *et al.*, 1977; Lopez *et al.*, 1978). Moreover, the loss of LAI activity after excision of the breast mass suggests that the source of the antigenic stimulus was confined to the lesion removed and not present, at least in enough quantity to evoke an immune response, in the remaining breast tissue which still contained fibrocystic disease. In all 5 patients, the LAI response of the LAI+ patient with BBD was identical to that for breast-cancer patients. Hence, the lesions of the LAI+ patients with BBD appear to express a similar TSA to that of unequivocal breast cancer. Although in some of the patients there was epithelial hyperplasia and atypia, histologically these changes did not amount to carcinoma *in situ*.

The biological implications of these findings are intriguing. It would appear that the breast tissue has undergone a transformation typical of carcinoma, without any morphological sign of unequivocal malignancy. Whether this implies an early, irretrievable step on the way to invasive carcinoma, or whether this change is reversible and is an expression of unusual but controlled cell proliferation cannot be resolved.

Certainly, it is known that patients with "benign" mammary dysplasia have a higher incidence of breast-cancer (Monson *et al.*, 1976; Haagensen, 1977; Donnelly *et al.*, 1975). Again, in the mouse angiogenesis, a property of carcinomas but not of resting mammary gland, appears earlier than any morphological evidence of malignancy in mouse papillomas which frequently progress toward invasive carcinoma (Brem *et al.*, 1977).

The present study showed that most patients with clinically detectable localized cancer (Stage I and II) manifested systemic antitumour immunity. Conversely, with the exception of a few patients of a select group of in-hospital patients with benign breast disease, most patients without breast cancer displayed no LAI activity to breast-cancer antigens and false-positive LAI responses were the result of experimental error, with an incidence of less than 5% against a single extract. By the use of a panel of breast-cancer antigens including testing the patient against her own tumour, it was possible to clarify, in most instances, false LAI+ or LAI− responses. Unfortunately, the use of a panel of antigens is too tedious to be used for routine testing.

This work was supported by the Medical Research Council of Canada, The National Cancer Institute of Canada and The Montreal Cancer Research Society Inc.

We thank the members of the Department of Surgery for allowing us to study their patients and Dr W. Duguid, Pathologist-in-Chief, for materials and pathology reports. We also thank Ms Mary Naughton for typing this manuscript.

REFERENCES

BLACK, M., BARCLAY, T., CUTLER, S. J., HANKEY, B. F. & ASIRE, A. T. (1972) Association of a typical characteristic of benign breast lesions with subsequent risk of breast cancer. *Cancer*, **29**, 338.

BREM, S. S., GULLINO, P. M. & MEDINA, D. (1977) Angiogenesis: A marker for neoplastic transformation of mammary papillary hyperplasia. *Science*, **195**, 880.

BURGER, D. R., VANDENBARK, A. A., FINKE, P., & 6 others (1977) Assessment of reactivity to tumor extracts by leukocyte adherence inhibition and dermal testing. *J. Natl Cancer Inst.*, **59**, 317.

BYERS, V. S., LEVIN, A. S., HACKETT, A. J. & FUDENBERG, H. H. (1975) Tumor speific cell-mediated immunity in household contacts of cancer patients. *J. Clin. Invest.*, **55**, 500.

DONNELLY, P. K., BAKER, K. W., CARNEY, J. A. & O'FALLON, W. M. (1975) Benign breast lesions and subsequent breast carcinoma in Rochester, Minnesota. *Mayo Clin. Proc.*, **50**, 650.

FAZEKAS, A. G. & MACFARLANE, J. K. (1977) Macromolecular binding of glucocorticoids in human mammary carcinoma. *Cancer Res.*, **37**, 640.

FAZEKAS, A. G., MARTI, J., FLORES, M., MACFARLANE, J. K. & THOMSON, D. M. P. (1978) Cortisol binding in human breast-cancer: correlation with antitumour immunity. *Cancer Immunol. Immunother.* (in press).

FLORES, M., MARTI, J. H., GROSSER, N., MACFARLANE, J. K. & THOMSON, D. M. P. (1977) An overview: antitumour immunity in breast cancer assayed by tube leucocyte adherence inhibition. *Cancer*, **39**, 494.

GRAHAM-POLE, J., OGG, L. J., ROSS, C. E. & COCHRAN, A. J. (1976) Sensitization of neuroblastoma patients and related and unrelated contacts to neuroblastoma extracts. *Lancet*, i, 1376.

GROSSER, N. & THOMSON, D. M. P. (1975) Cell-mediated antitumour immunity in breast cancer patients evaluated by antigen-induced leucocyte adherence inhibition in test tubes. *Cancer Res.*, **35**, 2571.

GROSSER, N. & THOMSON, D. M. P. (1976) Tube leucocyte (monocyte) adherence inhibition assay for the detection of anti-tumour immunity: III. "Blockade" of monocyte reactivity by excess free antigen and immune complexes in advanced cancer patients. *Int. J. Cancer*, **18**, 58.

HAAGENSEN, C. D. (1977) The relationship of gross cystic disease of the breast and carcinoma. *Ann. Surg.*, **185**, 375.

LOPEZ, M. J., O'CONNOR, R., MACFARLANE, J. K. & THOMSON, D. M. P. (1978) Natural history of anti-tumour immunity in human breast cancer assayed by tube leukocyte adherence inhibition. *Br. J. Cancer*, **38**, 660.

LOPEZ, M. J. & THOMSON, D. M. P. (1977) Isolation of breast cancer tumour antigen from serum and urine. *Int. J. Cancer*, **20**, 834.

LOWRY, O. H., ROSEBROUGH, N. J., FARR, A. L. & RANDALL, R. J. (1951) Protein measurements with the folin phenol reagent. *J. Biol. Chem.*, **193**, 265.

MARTI, J. H., GROSSER, N. & THOMSON, D. M. P. (1976) Tube leucocyte adherence inhibition assay for the detection of anti-tumour immunity. II. Monocyte reacts with tumour antigen *via* cytophilic anti-tumour antibody. *Int. J. Cancer*, **18**, 48.

MARTI, J. & THOMSON, D. M. P. (1976) Anti-tumour immunity in malignant melanoma assay by tube leucocyte-adherence inhibition. *Br. J. Cancer*, **34**, 116.

MONSON, R. R., YEN, S. & MACMAHON, B. (1976) Chronic mastitis and carcinoma of the breast. *Lancet*, ii, 224.

MORTON, D. L. & MALMGREN, R. A. (1968) Human osteosarcomas: Immunologic evidence suggesting an associated infectious agent. *Science*, **162**, 1279.

THOMSON, D. M. P. (1979) Antigens of breast cancer. In: *Methods in Cancer Research*. Ed. H. Busch. New York: Academic Press (in press).

THOMSON, D. M. P., GOLD, P., FREEDMAN, S. O. & SHUSTER, J. (1976) The isolation and characterization of tumor-specific antigens of rodent and human tumors. *Cancer Res.*, **36**, 3518.

THOMSON, D. M. P., RAUCH, J. E., WEATHERHEAD, J. C., & 5 others (1978) Isolation of human tumour-specific antigens associated with $\beta_2$ microglobulin. *Br. J. Cancer*, **37**, 753.